# Exploitation promotes earlier sex change in a protandrous patellid limpet, *Patella aspera* Röding, 1798

**DOI:** 10.1002/ece3.2925

**Published:** 2017-04-13

**Authors:** Gustavo M. Martins, Carla D. G. Borges, Maria Vale, Pedro A. Ribeiro, Rogério R. Ferraz, Helen R. Martins, Ricardo S. Santos, Stephen J. Hawkins

**Affiliations:** ^1^ CE3C ‐ Centre for Ecology, Evolution and Environmental Changes/Azorean Biodiversity GroupPonta DelgadaAzoresPortugal; ^2^ Department of BiologyUniversity of the AzoresPonta DelgadaAzoresPortugal; ^3^ Centre for Biological SciencesUniversity of SouthamptonSouthamptonUK; ^4^ CIIMAR ‐ Centro Interdisciplinar de Investigação Marinha e AmbientalPortoPortugal; ^5^ The Marine Biological Association of the United KingdomPlymouthDevonUK; ^6^ Ocean and Earth ScienceNational Oceanography Centre SouthamptonUniversity of SouthamptonSouthamptonHampshireUK; ^7^ MARE‐ Marine and Environmental Sciences CentreDepartment of Oceanography and Fisheries of the University of the AzoresHortaPortugal; ^8^ Regional Fisheries InspectionRegional Government of the AzoresHortaPortugal; ^9^ Department of Oceanography and Fisheries (DOP‐UAc)University of the AzoresAzoresPortugal

**Keywords:** conservation, gastropod, harvesting, Macaronesia, phenotypic plasticity, population structure, reproduction

## Abstract

Exploitation of organisms can prompt the reduction in the number and size of target populations consequently affecting reproductive output and replenishment. Here, we investigated the effects of exploitation on the population structure of a protandrous patellid limpet, *Patella aspera*, an overexploited Macaronesian endemic. Timed dives were used to collect animals across eleven islands of Macaronesia. Individuals were inspected for sex, size, and gonad stage. Using catch effort (time per person) per island coastal perimeter as a surrogate for exploitation intensity, we found that limpet abundance (CPUE) and mean size tended to decrease with exploitation intensity. When considering the sex of animals separately, the size of the largest male, but not females, decreased with exploitation. In contrast, the size of the smallest male remained relatively consistent, whereas the size of the smallest female decreased significantly with exploitation. As exploitation is mostly targeting larger individuals, results suggest that males are compensating the removal of larger females, by undergoing sex change at smaller and presumably earlier sizes. These results have wider implications for the conservation of *P. aspera*, as a reduction in female size will likely affect the numbers of oocytes produced, hence fecundity. Regulations promoting the protection of the larger‐sized animals should be enforced to safeguard the replenishment of the population.

## 1 Introduction

In a time when over 28% of all the world's fish stocks are either overexploited or depleted and when another 61% is fully exploited and in imminent danger of overexploitation (FAO 2014), it is paramount that the impact of fishing on the ecology of marine populations, communities, and ecosystems is better understood. Arguably, the largest anthropogenic impact in the Macaronesia intertidal is the overexploitation of patellid limpets (Hawkins, Allen, & Bray, 1999; Hawkins, Côrte‐Real, Pannacciulli, Weber, & Bishop, 2000); similar exploitation of limpets occurs in Hawaiian islands (e.g. McCoy, 2007). This has been taking place since Europeans first colonized these islands in the 15th century and in the case of the Canaries Islands since prehistoric times by the indigenous people. Remains of shells (middens) throughout the Canaries date back from the Guanche people, the first colonisers of the archipelago. Limpet harvesting in Macaronesia was moderate until the 1980s and was mainly for self‐consumption. However, introduction of snorkeling and scuba diving in the early 1980s together with the increase of the commercial value of limpets (e.g. tourism and exports to the USA) led to a prompt increase in limpet exploitation (Hawkins, Allen & Bray 1999; Ferraz, Menezes, & Santos, 2001).

Exploitation intensity varies among islands probably reflecting changes in human pressure (Martins, Jenkins, Hawkins, Neto, & Thompson, 2008). In the Azores, for instance, official statistics showed that in 1984, 94 tons were collected in São Miguel (where >50% of the Azorean population lives), while only 3 tons were collected across the whole of the remainder of the Azores (Martins, Santos, & Hawkins, 1987). Intense harvesting over the years contributed to the decline of limpets in the archipelago and the fishery collapsed in São Miguel in 1988 (Santos, Martins, & Hawkins, 1990).

The collapse in the stocks of limpets led to several investigations funded by the Azorean regional government (Martins et al., 1987; Santos, Hawkins, Monteiro, Alves, & Isidro, 1995; Santos et al., 1990), resulting in fishery regulations (e.g. D.R.R. 14/93/A Diário da República—I Série B, 178) for the collection of limpets. This regulation determined a limpet closed season (1st of October to 31st of May), and in the rest of the year, collection of limpets (above the established minimum sizes) was allowed outside protected areas to a maximum of 1 kg per person per day. Due to lack of enforcement of these management measures, limpet populations continued to suffer overexploitation during several years, particularly in the Central and Eastern island groups (Menezes, 1991; Ferraz, 1998; Ferraz et al., 2001; Martins, Thompson, Neto, Hawkins, & Jenkins, 2010; Martins et al., 2011; Santos et al. 1995) with the closed season now running between the 1st October and the 30th April (Portaria no. 1/2014, altered and republished by the Portaria no. 68/2016, 1st July 2016 and by Portaria n. 74/2015/A, 15th June 2015). Over the rest of the year, the collection of limpets is allowed, outside of the protected areas, above the established minimum sizes to a maximum of 80 kg per day per legal license holder, and a maximum of 1.5 kg for recreational harvesters on weekends and holidays. These new measures were setup taking into consideration current information on limpet populations, which show that, apart from high exploitation in specific areas, it is possible to maintain the commercial and recreational exploitation when restrictive rules are employed.

In the Canaries, the restricted distribution of the large intertidal limpet *Patella candei candei* d'Orbigny, 1840 to the Island of Fuerteventura, has also been linked to the impact of human harvesting (Côrte‐Real, Hawkins, & Thorpe, 1996; Weber & Hawkins, 2002; Núñez, Brito, Riera, Docoito, & Monterroso, 2003; but see González‐Lorenzo et al., 2015). It also occurs at the Portuguese Selvagens islands, where limited access and very strong protection ensure local conservation of this species. Other patellid limpets present in the Canary Islands, including *P. aspera*, also show clear signs of overexploitation (López et al., 2012; Navarro et al., 2005; Ramirez, 2009; Riera et al., 2016).

Exploitation of intertidal organisms rapidly prompts the reduction of the number and mean size of target populations (Castilla, 1999; Durán, Castilla, & Oliva, 1987; Griffiths & Branch, 1997; Hockey & Bosman, 1986; Lasiak, 1999; Moreno, Sutherland, & Jara, 1984; Oliva & Castilla, 1986). Both in the Azores (e.g. Martins et al., 2008) and in the Canaries (e.g. Navarro et al., 2005), significant reductions in abundance and size of limpets have been documented, especially in the most heavily exploited islands. The combined reduction in abundance and mean body size is likely to diminish the reproductive output of limpet populations. Moreover, size truncation of exploited populations may be of special concern in protandric hermaphrodite species, as these species reach sexual maturation as male then change to female later in life. So, the removal of larger animals will largely target females. *Patella aspera* in the Azores reaches sexual maturity between 41 and 45 mm shell length, with the peak of maximum gonad development being in January and the gonad resting period in May to June (Martins et al., 1987). While some species have a fixed size at sex change, such as the limpet *Cymbula oculus*, despite variation in environmental conditions (Munday, Buston, & Warner, 2006), other species have plastic responses (Fenberg & Roy, 2008; Rivera‐Ingraham, Espinosa, & García‐Gómez, 2011). Whether *P. aspera* has a fixed or plastic size at sex change is not known. Experimental harvesting in a congeneric protandric limpet *Patella vulgata* in the British Isles led to a decrease in shell size as well as in shell size at sex change (the size at which there is a 50:50 sex ratio), suggesting an earlier switch to females (Borges, Hawkins, Crowe, & Doncaster, 2016). Fisheries exploiting hermaphroditic species may disrupt sex ratios by skewing these to the sex that matures first, is smaller, and younger (Hamilton et al., 2007). Moreover, in sequential hermaphrodite species, size‐selective fishing can lead to sperm or egg limitation and reproductive failure in harvested populations (Alonzo & Mangel, 2004; Hamilton et al., 2007), particularly in species with a fixed sex change.

Understanding the rules governing sex change is a critical step for the proper management of exploited populations. For instance, while many management models include minimum size limits to prevent recruitment failure, management of sequential hermaphrodites, especially those with fixed size at sex change, may benefit from limits in which both the smaller and larger individuals are protected, thus preventing sperm or egg limitation and promoting higher fecundity (Alonzo & Mangel, 2004; Hamilton et al., 2007).

Here, we investigate how variation in human pressure across Macaronesian islands correlates with the population structure of the protandrous limpet *Patella aspera*. Although other patellid limpets can be found throughout the Macaronesia, we focus on *P. aspera* because: (1) it has a wide distribution occurring in all islands, (2) there is still debate regarding the taxonomic status of other patellid limpets (*P. candei* complex) (Côrte‐Real et al., 1996; Faria et al., 2016; Sá‐Pinto, Branco, Harris, & Alexandrino, 2005; Weber & Hawkins, 2002), and (3) it is the most exploited species with the highest economical value (Martins, 2009; Martins et al., 1987; Navarro et al., 2005). We analyzed data collected from 12 islands (eight from the Azores and four from the Canary Islands) to test the hypotheses that increasing human pressure is associated with reductions in limpet abundance and size. We further predict that if size at sex change is fixed in *P. aspera*, sex ratio will be highly skewed toward male (smaller individuals) in the most exploited islands. In contrast, if size at sex change is plastic in *P. aspera*, sex ratio may remain relatively unchanged provided that the size at sex change decreases with increasing exploitation.

## 2 Materials and methods

### 2.1 Data collection

Limpets were collected in each of the twelve islands by the same experienced harvesters during standard 30‐min dives. The number and date of samples (dives) varied among islands according to opportunity (summarized in Table 1). All animals were measured (shell length) using calipers and dissected to inspect the gonads. Individuals were sexed and staged according to Orton, Southward, and Dodd (1956). Males and females are easily identified by the color of the gonads (pale white or pink in males, brown to red in females), while individuals without conspicuous sex characteristics (generally smaller animals or animals collected during the summer resting season (Martins et al., 1987; Vale, 2016)) were classified as neuter. For each island, we calculated: mean capture per unit of effort (CPUE: number of animals caught in 30 min dives) and mean percentage of females as well as the maximum and minimum size attained by males and females on any sample.

**Table 1:** Summary of samples of limpets collected by experienced harvesters during 30‐min dives

Archipelago	Island	Sampling year	# dives (samples)	# individuals	Index of exploitation
Azores	Corvo	89	2	351	0.03
	Flores	87–89	7	672	0.08
	São Jorge	87	2	64	0.11
	Santa Maria	89	5	693	0.14
	Pico	86–03	43	4,321	0.17
	Faial	86–98	39	4,883	0.32
	Terceira	87–89	13	628	0.79
	São Miguel	86–04	16	489	1.00
Canaries	La Gomera	92	2	210	0.09
	Fuerteventura	92	5	282	0.10
	La Palma	92	6	100	0.22
	Tenerife	92	13	444	0.74

### 2.2 Index of exploitation and data analysis

Official landing records were found to be highly unreliable in the case of the limpet fishery where a large proportion of the catches were directly sold to restaurants or on the street (Martins, 2009) and/or are illegally collected (Martins et al., 2011). Instead, Martins et al. (2008) found that an approximate index of exploitation could be calculated using the number of people and the coastal perimeter of each island. This index provides an overall index of human pressure and was found to correlate well with the numbers (density) of large limpets in the Azores (Martins et al., 2008), indicating that it may be particular useful as an index of exploitation. Similar indices (i.e., numbers of fishing boats per island) have also been successfully used to assess the impact of fisheries in the Canary Islands (e.g., Tuya, Sanchez‐Jerez, & Haroun, 2006). The index of exploitation was hence calculated by dividing the number of people per coastal perimeter. For clarity, the index was then scaled to ≤1, where 1 indicates the island with the highest exploitation index.

Spearman rank correlation was used to test the null hypothesis that the index of exploitation was not correlated with the variables of interest. The following variables were tested: mean abundance (CPUE) per island, mean limpet size, mean percentage females per island, maximum size of males, maximum size of females, and male‐to‐female minimum size ratio. The later, together with the analysis of size at which males and females reached sexual maturity, was used to investigate whether changes in sex occurred in a block (males turn into females at smaller sizes, but so do neuters into males) or unevenly (e.g., males turn into females at smaller sizes, but neuters do not turn into males at smaller sizes). The later, if correlated with exploitation, would be an indication that males would be undergoing sex change at smaller sizes to somehow compensate the removal of females from the system and would not be the result of reaching sexual maturity, as a whole (males and females) at smaller sizes.

## 3 Results

**Figure 1:**
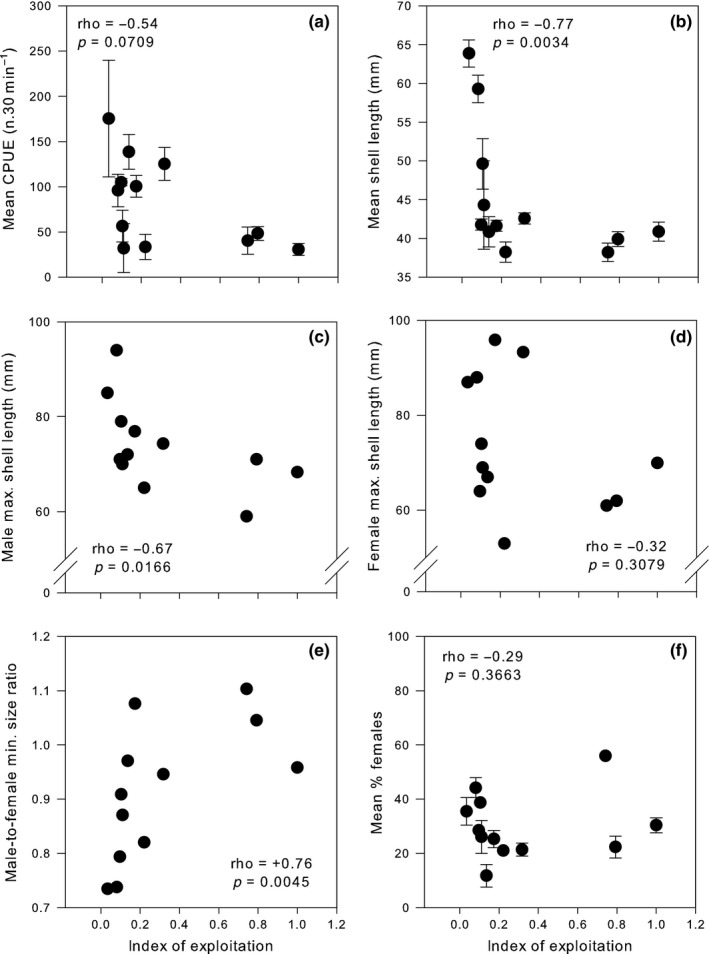
Scatter plots showing the relationship between the index of exploitation and (a) mean (±SE) limpet CPUE, (b) mean (±SE) animal shell size, (c) male maximum shell size, (d) female maximum shell size, (e) male‐to‐female minimum shell size ratio, and (f) mean (±SE) percentage of females. Each point represents one island. The Spearman rank correlation (rho) between the index of exploitation and variables examined is presented in each plot

The abundance of *P. aspera* (CPUE) tended (*p *= .07) to be progressively and negatively correlated with exploitation pressure (Figure 1a). Mean animal size was negatively correlated with exploitation (Figure 1b), but unlike abundance, the reduction in mean size was nonlinear reducing abruptly at moderate levels of exploitation (Figure 1b). Size of the largest male collected was negatively correlated with the index of exploitation (Figure 1c) with a 20% reduction in shell length between the least and most exploited islands. Unlike males, the size of the largest female did not correlate with the index of exploitation (Figure 1d). Moreover, when considering patterns of minimum shell length, the male‐to‐female ratio was positively correlated, and significantly so, with the index of exploitation (Figure 1e). Separate analyses of the size of the smallest male and female *P. aspera* showed that whereas both tended to correlate negatively with the index of exploitation (male: rho = −0.52, *p *=* *.09, female: rho = −0.75, *p *<* *.01), the reduction in size in females was much more pronounced. This suggests that limpets probably changed sex from male to female at smaller sizes with increased exploitation. The mean percentage of females in the population remained relatively constant among islands and did not correlate with the index of exploitation, suggesting that size at sex change is not fixed (Figure 1f).

## 4 Discussion

Many locations worldwide with long‐established human populations have a tradition of local exploitation of marine resources including those in the littoral (Siegfried, 1994). Such exploitation can be particularly intense on islands (Erlandson & Rick, 2010) leading to overexploitation of stocks and, in extreme cases, local extinction of species (e.g., *Patella candei* in Lanzarote, Weber & Hawkins, 2002). The shores of Macaronesia are one such example, as they have been heavily exploited for food since the islands were first colonized by people (Hawkins et al., 2000) and many stocks including lobsters, barnacles, trochids, and limpets are currently overexploited (Morton, Britton, & Martins, 1998; Santos et al., 1995).

### 4.1 Direct effects of limpet harvesting

A large body of evidence has shown that harvested populations of intertidal gastropods often show simultaneous reduction in the numbers and size of individuals (e.g., Branch, 1975; Castilla, 1999; Lasiak, 1993, 1999; Moreno et al., 1984). Our results are in line with the above, as both the numbers and size of *P. aspera* decreased with exploitation (although for numbers the relationship was not significant at α = 0.05, see results for further details). While the reduction in abundance tended to be linear with exploitation, the reduction in animal size was much more abrupt. This result confirms our perception that limpet fishery is highly size‐selective targeting preferentially the larger animals. Moreover, it also suggests that *P. aspera* may be directly more susceptible to size exploitation (reduction in size) than recruitment exploitation (reduction in the numbers of animals of harvestable size) per se. This is important not only for the population dynamics of the species (e.g., reproduction) but may also have wider community‐level impacts as both animal size and density additively affect the activity in intertidal snails (e.g., Atkins, Griffin, Angelini, O'Connor, & Silliman, 2015; Martins, Prestes, & Neto, 2013).

### 4.2 Impact of harvesting on sex change

Results showed that size of *P. aspera* generally decreased with exploitation. When considering the sex of animals separately, results showed that the size of the largest male, but not females, decreased with exploitation. In contrast, the size of the smallest male remained relatively consistent throughout the exploitation gradient, whereas the size of the smallest female decreased significantly with exploitation. As exploitation is mostly targeting the larger individuals (see Figure 1b), results suggest that males are compensating the removal mainly of the larger females, by undergoing sex change at smaller and presumably earlier sizes. That the size of the smallest male has remained relatively stable throughout the archipelago also suggests that the switch of neuters into males has not been affected (in contrast to Borges et al., 2016 in *P. vulgata*). These results suggest that the numbers of males in the population must have been reduced in the most exploited islands. That the proportion of females also remained consistent across the gradient of exploitation suggests that compensation is occurring due to early progression of males to females when the later are removed by harvesting. These results are also very similar to those found by Borges et al. (2016) for *P. vulgata* in the British Isles.

The mechanism triggering sex change in *P. aspera* remains to be explored. It could be simple a density‐dependent regulation in function of some limiting resource (e.g., food), that is, reduced densities leading to greater food resources, so that more energy can be invested in expensive egg production prompting sex change (Wright, 1989). However, intraspecific competition in the limpet *P. depressa* was shown to be asymmetric among size classes, with larger animals having a much greater negative impact on the growth and mortality of individuals of the smaller size classes (Boaventura, Fonseca, & Hawkins, 2003). If this is also the case in *P. aspera*, then regulation of size at sex change may not be simply a function of animal density, and the specific presence of large individuals may have a more direct role. Again, this suggests that the effects of size exploitation may differ from the effects of recruitment exploitation. Thus in addition to inter‐age class interactions found in gonochoristic *Patella depressa,* inter‐age and size class interactions are probably between sexes in protandric species.

### 4.3 Implications for conservation

Populations of *P. aspera* appear to be more susceptible to size exploitation than recruitment exploitation, whereby larger animals, mostly females, are preferentially harvested from the population. Like in other patellid limpets (Borges et al., 2016; Rivera‐Ingraham et al., 2011), *P. aspera* appears to adapt with males undergoing sex change into females earlier and thus compensating for the reduced numbers of females. While this mechanism appears to be able to compensate for the relative numbers of males and females in the population across the gradient of exploitation, its wider consequences for reproductive output remain to be tested. Size of females in patellid limpets does not seem to affect the size of oocytes produced, but does affect the overall numbers of oocytes produced, hence fecundity (Espinosa, Guerra‐García, Fa, & Gárcia‐Gómez, 2006). This may, in turn, lead to reproductive failure. In fact, Claereboudt (1999) simulated the effects of spatial distribution, rate of gamete release, and population structure (density and sex ratio) on fertilization success and found that even a mild fishing pressure could lead to dramatic (90%) reductions in larval production. Inbreeding coefficients (F_IS_) for *P. aspera* in the Macaronesia (Faria et al., 2016) do suggest a relative small effective population size, which could be an indication of the lack of large females as a result of size exploitation. Moreover, the latter (Faria et al., 2016) also showed significant genetic variation between populations of *P. aspera* among Macaronesian archipelagos, which is of applied interest for the definition and management of limpet stocks (Hawkins et al., 2016).

Considering the above, we suggest that on top of current legislation defining minimum sizes, legislation promoting the protection of the larger‐sized individuals (females), such as the establishment of maximum sizes, should also be passed and fully enforced throughout the Macaronesia. This would allow the survival of a greater number of large animals and potentially promoting an increase in the effective population size.

## Authors' contributions

S.J.H., H.R.M., and R.S.S. conceived the idea and designed the methodology; C.D.G.B., M.V., P.A.R., R.R.F., H.R.M., R.S.S., and S.J.H. collected data; S.J.H. and G.M.M. devised the hypotheses with G.M.M analyzing the data; G.M.M. led the writing of the manuscript. All authors contributed critically to the drafts and gave final approval for publication.

## Conflict of interest

None declared.

## Data accessibility

Data will be made publicly available from Dryad if the manuscript is accepted for publication.
